# Incidence and persistent infection rates of type-specific HPV among HIV-infected males in China: a 5-year retrospective study

**DOI:** 10.3389/fpubh.2025.1690348

**Published:** 2025-11-05

**Authors:** Siwei Zhang, Huan Liu, Zhiyan Huang, Siyu Duan, Yuting Zhang, Yanjing Li, Wenzhu Chu, Yan Wang, Rongqing Yang, Lanlan Wei

**Affiliations:** ^1^National Clinical Research Center for Infectious Diseases, The Third People's Hospital of Shenzhen, The Second Hospital Affiliated to Southern University of Science and Technology, Shenzhen, China; ^2^Department of Endodontics, The First Affiliated Hospital of Harbin Medical University, School of Stomatology, Harbin Medical University, Harbin, China; ^3^School of Public Health, Guangzhou Medical University, Guangzhou, China; ^4^Laboratory of Medical Genetics, Harbin Medical University, Harbin, China; ^5^Department of Dermatology and Venereology, The Third People's Hospital of Shenzhen, The Second Hospital Affiliated to Southern University of Science and Technology, Shenzhen, China; ^6^Department of Microbiology, Harbin Medical University, Harbin, China

**Keywords:** human papillomavirus, HPV vaccines, HIV, men who have sex with men, sexually transmitted diseases

## Abstract

**Background:**

Although the 9-valent HPV vaccine (9v-HPV) has been approved for males in China, it has received limited public attention. This study aimed to explore the incidence and persistence of different HPV types in HIV-related males and emphasize the importance of vaccination.

**Methods:**

A cohort of 758 male patients with multiple HPV detection (≥3) from 2018 to 2023 in our hospital were enrolled for statistical analysis, including 113 Men who have Sex with Men (MSM) patients. HIV and HPV infection statuses were confirmed respectively by ELISA, PCR, WB and nucleic acid kit.

**Results:**

Statistical analysis revealed that the incidence of 9v-HPV-covered high-risk (HR) HPV infections were 68.1% (HIV^+^ total males), 43.2% (HIV^−^ total males), 67.6% (HIV^+^ MSM) and 42.2% (HIV^−^ MSM). HPV persistence was significantly prolonged in HIV^+^ patients than HIV^−^ cases (*p* < 0.001). Markov model demonstrated that HIV increased incidence risks of HPV52, 58, 45, 35, 39, 51, 59, 68, 66, 73, 82, 81 and reduced the clearance rates of HPV16, 18, 6, 11, 58, 33, 31, 56 in males.

**Discussion:**

These findings highlight the increased burden of HPV infection among HIV^+^ individuals. Timely vaccination will benefit males by preventing HPV infection and consequently reducing the associated disease burden.

## Introduction

Persistent human papillomavirus (HPV) infection is associated with the development of various clinical manifestations including condyloma acuminatum, anogenital cancers, and head and neck malignancies (1, 2). More than 200 HPV genotypes have been identified, which are categorized into high-risk HPV (HR-HPV) and low-risk HPV (LR-HPV) based on their oncogenic potential (3, 4). Epidemiological surveillance data indicate that while HPV vaccination efforts have resulted in decreased infection rates in recent decades, the incidence of HPV-associated cancers continues to rise (5).

Global meta^−^analytic data indicate that approximately 1/3 of the male population worldwide has been exposed to HPV infection (6). People living with Human Immunodeficiency Virus (HIV) and men who have sex with men (MSM) exhibit significantly higher susceptibility to HPV acquisition and a greater disease burden related to HPV infection (7–9). Regional epidemiological studies in China reveal an HPV prevalence of 62.8% among MSM, with a significantly higher rate of 82.7% observed in HIV^+^ MSM populations (10). Clinical observations demonstrate that during persistent HPV infection, patients may acquire of novel HPV genotypes or experience spontaneous clearance of existing infections (11). Among HIV^+^MSM, the incidence of anal HR-HPV infection is increased while clearance rates are reduced (12). However, the impact of HIV on the incidence, persistence, and clearance of different HPV genotypes in male populations has not been reported.

Currently available HPV vaccines primarily include bivalent (2v-HPV), quadrivalent (4v-HPV), and 9-valent vaccine (9v-HPV), which are mainly used for cervical cancer prevention in females (2, 13). 9v-HPV comprises 7 HR-HPV (HPV16, 18, 31, 33, 45, 52, 58) and 2 LR-HPV (HPV6, 11) genotypes. In 2011, the United States became the first country to incorporate routine male HPV vaccination into its national immunization program, including MSM up to age 26 years. Vaccination has demonstrated effectiveness in reducing HPV infections among MSM (14). In 2025, China approved its first and only HPV vaccine licensed for male use. Given the documented geographic variations in HPV genotype distribution (6), the potential protective efficacy of HPV vaccines for male populations in China requires further evaluation.

The current understanding of HPV infection in male remains constrained by the predominant reliance on cross-sectional study designs, which fundamentally limit the accurate estimation of HPV incidence rates and clearance probabilities. To address these critical knowledge gaps, we systematically analyzed 20 HPV genotypes in male patients who underwent three or more HPV tests at Shenzhen Third People’s Hospital between 2018 and 2023. Our study comprehensively evaluated the incidence and persistence rates of various HPV genotypes among both HIV^+^ and HIV^−^ male patients, with further analysis of the determinants driving transitions between distinct HPV infection states. These findings showed men living with HIV experienced a greater burden of HPV-related disease and emphasized the critical role of vaccination in HPV prevention in HIV^+^ males, while simultaneously provided empirical support for the development of targeted HPV vaccination strategies for males in China.

## Materials and methods

### Study design and data collection

**Figure 1:**
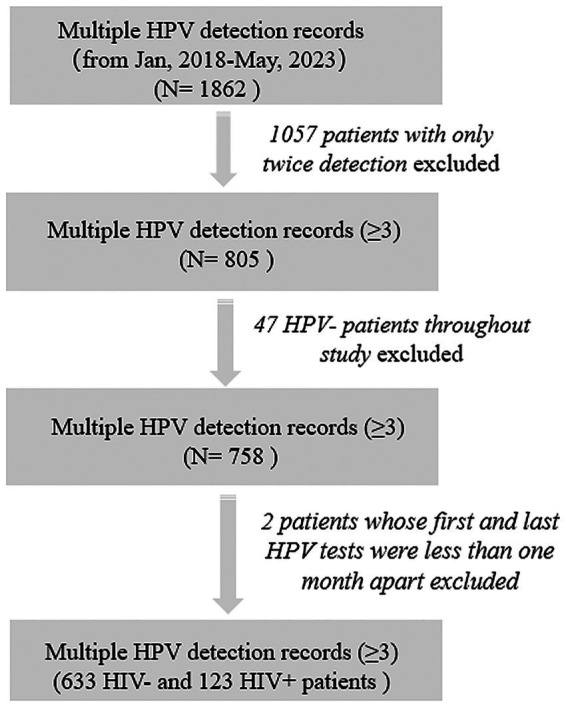
Screening flowchart for inclusion of records.

This study was a retrospective cohort study conducted in The Third People’s Hospital, Shenzhen, China. The study complied with the ethical principles of the Declaration of Helsinki and its design and protocol have been reviewed and approved by the hospital ethics committee (2022–102-05). All patient data were anonymized and handled with strict confidentiality to safeguard privacy. To assess the persistence duration of HPV infection and ensure statistical precision, all male patients with multiple HPV detection (≥3) from 2018 to 2023 were included in the cohort. Most of these patients performed HPV testing due to reasons such as “high-risk sexual behaviors,” “presentation of typical clinical symptoms,” or “having sexual partners with sexually transmitted diseases (STDs),” thus being identified as a high-risk population for HPV. We further excluded patients who had consistently negative HPV test results across all tests. For patients with only three tests, the intervals between HPV tests should exceed 1 month (Figure 1). HPV detection swabs were collected from the perianal, anal, or penile areas of the patients. Demographic characteristics (age) and other clinical data (sexual behavior, HIV infection state) were obtained by reviewing electronic medical records of patients. Patients who self^−^reported engaging in “anal sex” or “male-to-male sexual contact” were defined as MSM population. After HPV infection, physicians developed treatment plans based on the clinical symptoms. The primary therapeutic approaches include the treatment plan included local therapy tailored to the location of the patient’s skin lesions, along with systemic treatments aimed at combating HPV and enhancing the immune system. For patients co-infected with HIV, anti-HIV treatment was administered concurrently.

### Human papillomavirus detection

According to the instructions, 20 HPV types were identified by HPV nucleic acid Typing Kit (BioPerfectus Technologies, China) According to the instructions, including HPV16, 18, 31, 33, 35, 39, 45, 51, 52, 56, 58, 59, 68, 66, 82, 53, 73, 11, 6 and 81.

### HIV infection diagnostic

For patients with high-risk exposure more than 7 days prior, initial testing was performed using ELISA for HIV-1/2 antibodies and PCR for HIV nucleic acid. If the results were discordant, repeat testing was conducted using PCR and Western blot (WB) for nucleic acid and HIV-1/2 antibody confirmation. If hospital^−^based testing consistently indicated possible HIV infection, the sample was referred to the CDC (Center for Disease Control and Prevention) for confirmatory testing in accordance with relevant regulations, ultimately determining the patient’s HIV infection status.

### Definitions of HPV infection persistence and clearance

The persistence of HPV infection was defined as the period from the first positive test to the last positive test for a given HPV subtype. HPV clearance was strictly defined as two consecutive negative test results (1–0-0) with an interval of >1 month between tests. True viral clearance required two sequential negative results following a positive baseline (1–0-0). HPV incidence were specifically analyzed among baseline HPV^+^ patients, with new infections defined by seroconversion from negative to positive status (0–1) during follow-up. A transient negative result followed by a positive test (1–0-1) was not considered clearance, as it may reflect intermittent detection due to low viral copy numbers below the assay’s limit of detection rather than true eradication. Each HPV infection was treated as an independent event in the analysis.

### Markov model construction

Given the observed intermittent transitions between negative and positive test results in some individuals, we implemented a Markov model to characterize dynamic HPV infection state transitions (15), specifically modeling bidirectional conversion processes encompassing negative^−^to^−^positive and positive-to-negative transitions. The model quantified state transition rates by calculating annualized instantaneous transition intensity. Transition intensities were estimated by maximum likelihood, incorporating person-years of observation and accounting for intermittent sampling intervals. Each HPV infection episode was treated as an independent observation event. The model was employed to estimate transition rates between HPV infection states while assessing covariate effects through hazard ratios (HRs) with 95% confidence intervals (95% CIs).

### Statistical analysis

Chi-square test was utilized to compare categorical variables between groups, with Pearson’s chi^−^square statistic calculated for contingency tables meeting the expected cell frequency assumption (>5 in all cells); when this assumption was violated (≥20% of cells with expected counts <5), Fisher’s exact test was employed instead to compute the exact probability of observed distributions in 2 × 2 contingency tables. Mann^−^Whitney U test was conducted as a nonparametric alternative to compare two independent groups. All analyses were performed using R statistical software (version 4.4.1). *, *p* < 0.05.

## Results

### Participant characteristics

This study enrolled 756 high-risk male patients with HPV infection who had visited the hospital at least three times between 2018 and 2023, including 123 HIV^+^ and 633 HIV^−^ individuals. Based on self^−^reported history of male-to-male sexual contact, a total of 113 men who have sex with men (MSM) were identified, comprising 68 HIV^+^ and 45 HIV^−^ cases.

The mean age of all HIV^+^ total males was 31.23 years (IQR: 26.00–34.00 years), while that of HIV^−^ total males was 32.86 years (IQR: 27.00–38.00 years). The majority of HPV patients in the our cohort were under 35 years old. However, the average age of HIV^+^ MSM was higher than that of their HIV^−^ MSM (Tables 1, 2). A finer age stratification demonstrated that most HPV^+^ cases were clustered in the 27 ~ 35 year old range (Supplementary Table 1). The mean follow-up duration was 1.64 years (IQR: 0.69–2.24) for HIV^+^ male patients, 1.31 years (IQR: 0.56–1.73) for HIV^−^ patients.

**Table 1:** Basic clinical information of total males.

Characteristics	Total (N = 756)		*χ* ^2^	*p*-value^a^
	HIV^+^(N = 123)	HIV^−^(N = 633)		
Age (years)				
Average (IQR^b^)	31.23 (26.00–34.00)	32.86 (27.00–38.00)		
			7.85	0.01*
<35	93 (0.76)	395 (0.62)		
≥35	30 (0.24)	238 (0.38)		
Clinical visits (times)				
Average (IQR)	4.55 (3.00–5.00)	4.59 (3.00–5.00)		
			0.19	0.66
<5	82 (0.67)	409 (0.65)		
≥5	41 (0.33)	224 (0.35)		
Follow-up duration (years)				
Average (IQR)	1.64 (0.69–2.24)	1.31 (0.56–1.73)		
			6.61	0.04*
<1	50 (0.41)	322 (0.51)		
1 ~ 2	36 (0.29)	182 (0.29)		
≥2	37 (0.30)	129 (0.20)		

a *p*-value were calculated by Chi-sq. test.

b IQR, Interquartile range.

**Table 2:** Basic clinical information of MSM.

Characteristics	MSM (N = 113)		*χ* ^2^	*P*-value^a^
	**HIV** ^**+**^ **(N = 68)**	**HIV** ^**−**^ **(N = 45)**		
Age (years)				
Average (IQR^b^)	31.34 (26.00–34.00)	28.31 (24.00–31.00)		
			1.06	0.30
<35	52 (0.76)	38 (0.84)		
≥35	16 (0.24)	7 (0.16)		
Clinical visits (times)				
Average (IQR)	4.72 (3.00–5.00)	4.49 (3.00–5.00)		
			0.08	0.77
<5	42 (0.62)	29 (0.64)		
≥5	26 (0.38)	16 (0.36)		
Follow-up duration (years)				
Average (IQR)	1.67 (0.73–2.53)	1.15 (0.58–1.48)		
			4.29	0.12
<1	25 (0.37)	24 (0.53)		
1 ~ 2	22 (0.32)	14 (0.31)		
≥2	21 (0.31)	7 (0.16)		

a *p*-value were calculated by Chi-sq. test.

b IQR, Interquartile range.

**Figure 2:**
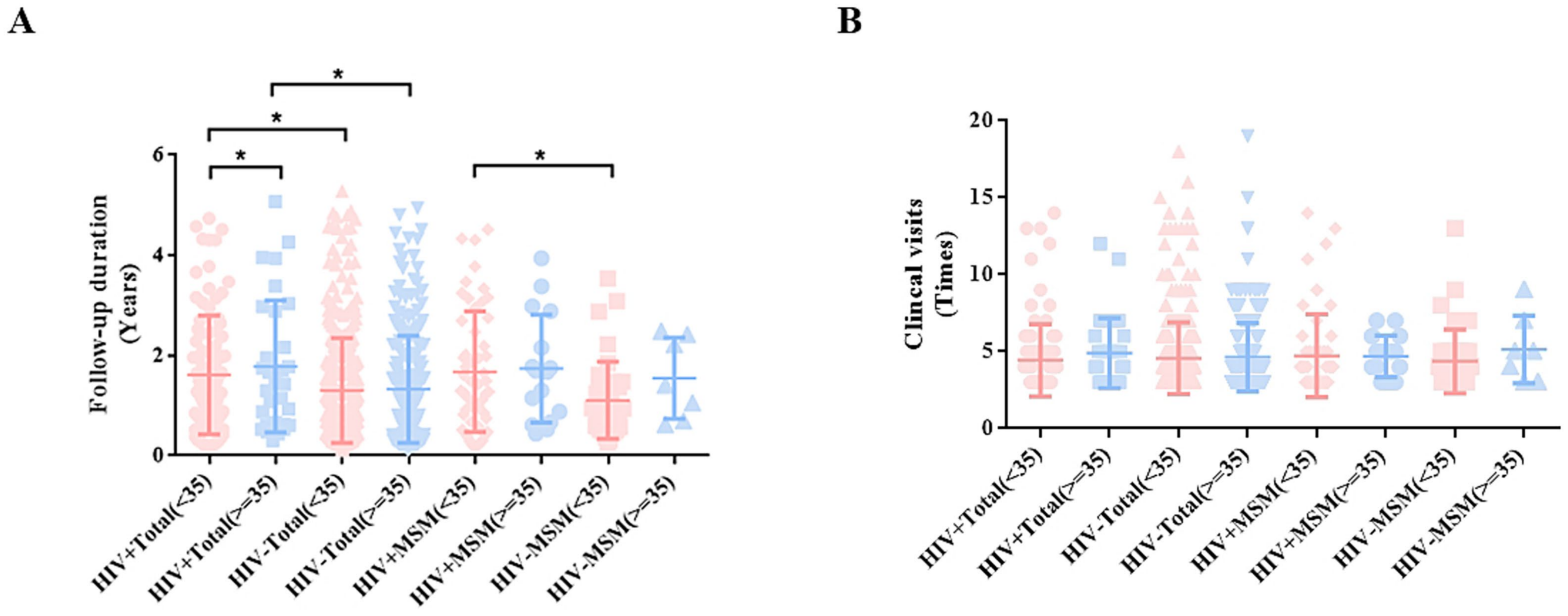
Analysis of the correlation between patient age, follow-up duration, and number of clinical visits. **(A)** Follow-up duration among patients in different groups. **(B)** Clinical visit times among male patients in different groups.

Comparison of follow-up duration across age groups revealed that HIV^+^ total males (aged ≥35 years) years had a significantly longer duration than younger patients (aged <35 years) (*p* < 0.05). Similarly, among MSM (aged <35 years), HIV^+^ MSM had significantly prolonged follow-up duration compared to HIV^−^ individuals (*p* < 0.05) (Figure 2A). However, no significant association was observed between the number of clinical visits and either HIV status or age (Figure 2B).

### Baseline characteristics of type-specific HPV infection in male patients

A baseline analysis of 673 patients with initial HPV infection revealed distinct patterns by HIV status. Among HIV^+^ total males, the proportion of multiple HPV infections significantly exceeded single infections (78% vs. 22%), with a similar trend observed for HR-HPV infections (71% vs. 29%). In contrast, HIV^−^ total males showed no significant disparity in single versus multiple infections for either overall HPV (47% vs. 53%) or HR-HPV (46% vs. 54%).

Notably, both HIV^+^ and HIV^−^ males showed a higher prevalence of single LR-HPV infections compared to multiple LR-HPV infections (*p* < 0.001). Among HIV^+^ MSM, multiple HPV (82%) and HR-HPV (77%) infections predominated, whereas HIV^−^ MSM demonstrated no such trend. Conversely, single LR-HPV infections were more common in both HIV^+^ (71%) and HIV^−^ (83%) MSM. HIV infection was significantly associated with increased multiple HPV and HR-HPV infections in total male patients and MSM subgroup (*p* < 0.001) (Tables 3, 4).

**Table 3:** Baseline prevalence of high-risk vs. low-risk HPV infections in total males.

Characteristics	HIV^+^ total	HIV^−^ total	*χ* ^2^	*p-*value^a^
Any HPV			36.41	0.00*
Single infection	25 (0.22)	298 (0.53)		
Multiple infection	88 (0.78)	262 (0.47)		
HR-HPV			19.10	0.00*
Single infection	27 (0.29)	196 (0.54)		
Multiple infection	66 (0.71)	164 (0.46)		
LR-HPV			13.06	0.00*
Single infection	70 (0.74)	346 (0.88)		
Multiple infection	25 (0.27)	46 (0.12)		

a *p*-value were calculated by Chi-squared test.

**Table 4:** Baseline prevalence of high-risk vs. low-risk HPV infections in MSM.

Characteristics	HIV^+^MSM	HIV^−^MSM	*χ* ^2^	*p-*value^a^
Any HPV			12.57	0.00*
Single infection	12 (0.18)	22 (0.49)		
Multiple infection	56 (0.82)	23 (0.51)		
HR-HPV			11.76	0.00*
Single infection	13 (0.23)	15 (0.63)		
Multiple infection	44 (0.77)	9 (0.38)		
LR-HPV			1.66	0.20
Single infection	42 (0.71)	33 (0.83)		
Multiple infection	17 (0.29)	7 (0.18)		

a *p*-value were calculated by Chi-squared test.

**Figure 3:**
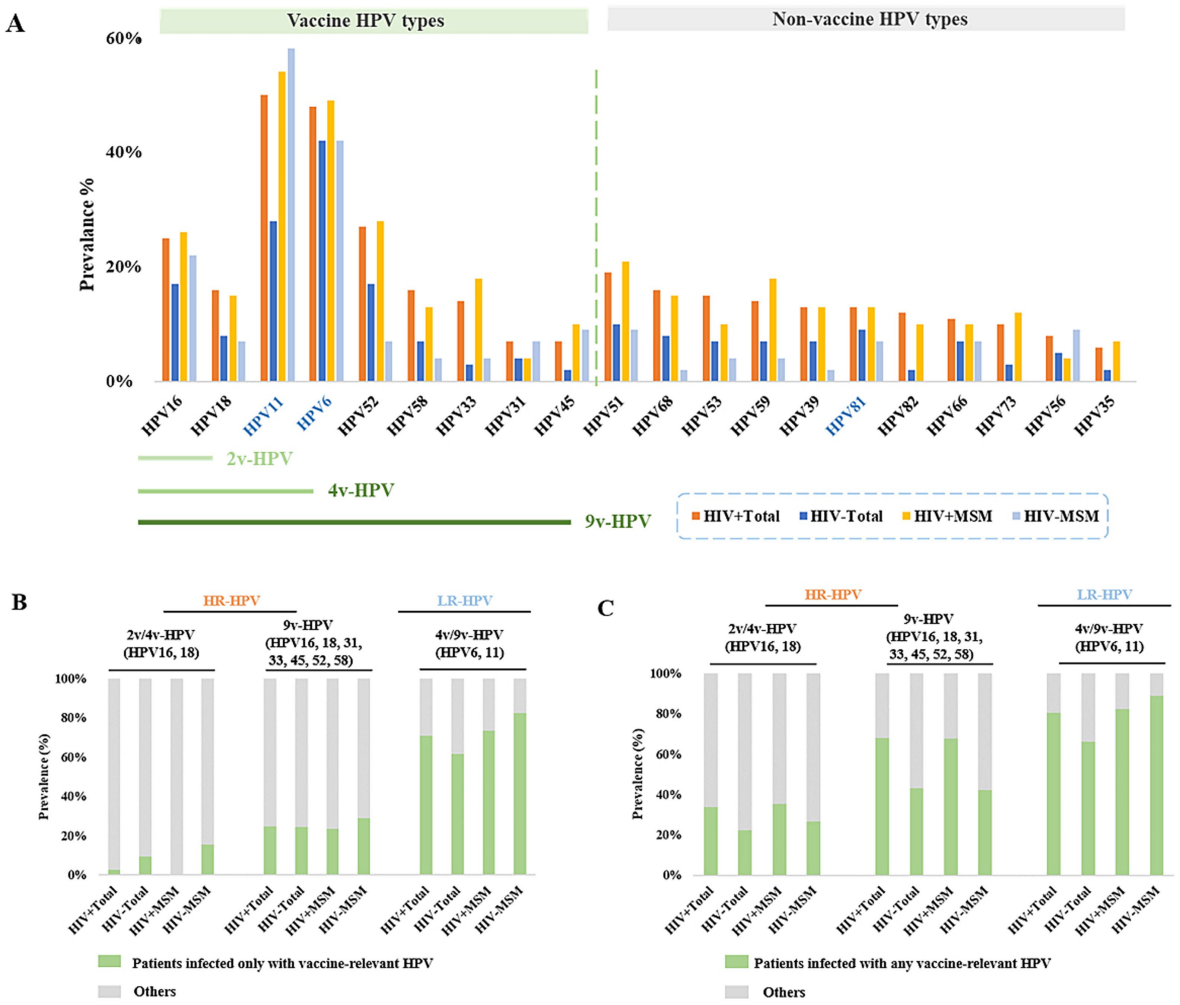
Prevalence of different HPV types among male patients at initial clinical presentation. **(A)** Prevalence of vaccine-targeted and non-vaccine-targeted HPV types in HIV^+^ and HIV^−^ male cohorts. **(B)** Prevalence of patients infected only with vaccine-relevant HPV. **(C)** Prevalence of patients infected with any vaccine-relevant HPV.

Analysis of the initially detected HPV genotypes revealed distinct prevalence patterns. Among HIV^+^ total males, the most prevalent HR-HPV types were HPV52 (27%), HPV16 (25%), HPV51 (19%), and HPV58 (16%); in HIV^−^ total males, the highest^−^prevalence HR-HPV types were HPV52 (17%), HPV16 (17%), HPV51 (10%), HPV68 (8%), and HPV18 (8%). Among HIV^+^MSM, the predominant HR-HPV types included HPV52 (28%), HPV16 (28%), HPV51 (21%), HPV59 (18%), and HPV33 (18%). HIV^−^ MSM showed HPV16 (22%), HPV51 (9%), HPV45 (9%), and HPV56 (9%) as the most prevalent HR-HPV types (Figure 3A; Supplementary Table 2).

We then analyzed the incidence of genotype-specific HPV targeted by the HPV vaccines. The results revealed that only 2.7% of HIV^+^ total males, 9.5% of HIV^−^ total males had HPV16 and/or HPV18 infections without coinfection with other detectable HR-HPV types. We subsequently examined infections caused by HR-HPV types covered by the 9v-HPV, including HPV16, 18, 31, 33, 45, 52, and 58. A similar prevalence was observed for these HR-HPV infections in different groups: HIV^+^ total males (24.8%), HIV^−^ total males (24.3%), HIV^+^MSM (23.5%), and HIV^−^ MSM (28.9%). The prevalence of HPV6 and/or HPV11 infection were 70.8% in HIV^+^ total patients, 61.4% in HIV^−^ total males, 73.5% in HIV^+^ MSM, and 82.2% in HIV^−^ MSM (Figure 3B; Supplementary Table 3S1). We further assessed the prevalence of patients with 9v-HPV-targeted HR-HPV, with or without co-infection with other HR-HPV types. We found that the prevalence of infection with 9v-HPV-related HR-HPV types was 68.1% in HIV^+^ total males, 43.2% in HIV^−^ total males, 67.6% in HIV^+^MSM, and 42.2% in HIV^−^MSM (Figure 3C; Supplementary Table 3). These findings suggested that the majority of HPV infections in the male population were covered by the 9-valent vaccine, demonstrating its potential efficacy in preventing HPV-related disease among men.

### Duration of type-specific HPV persistence

**Figure 4:**
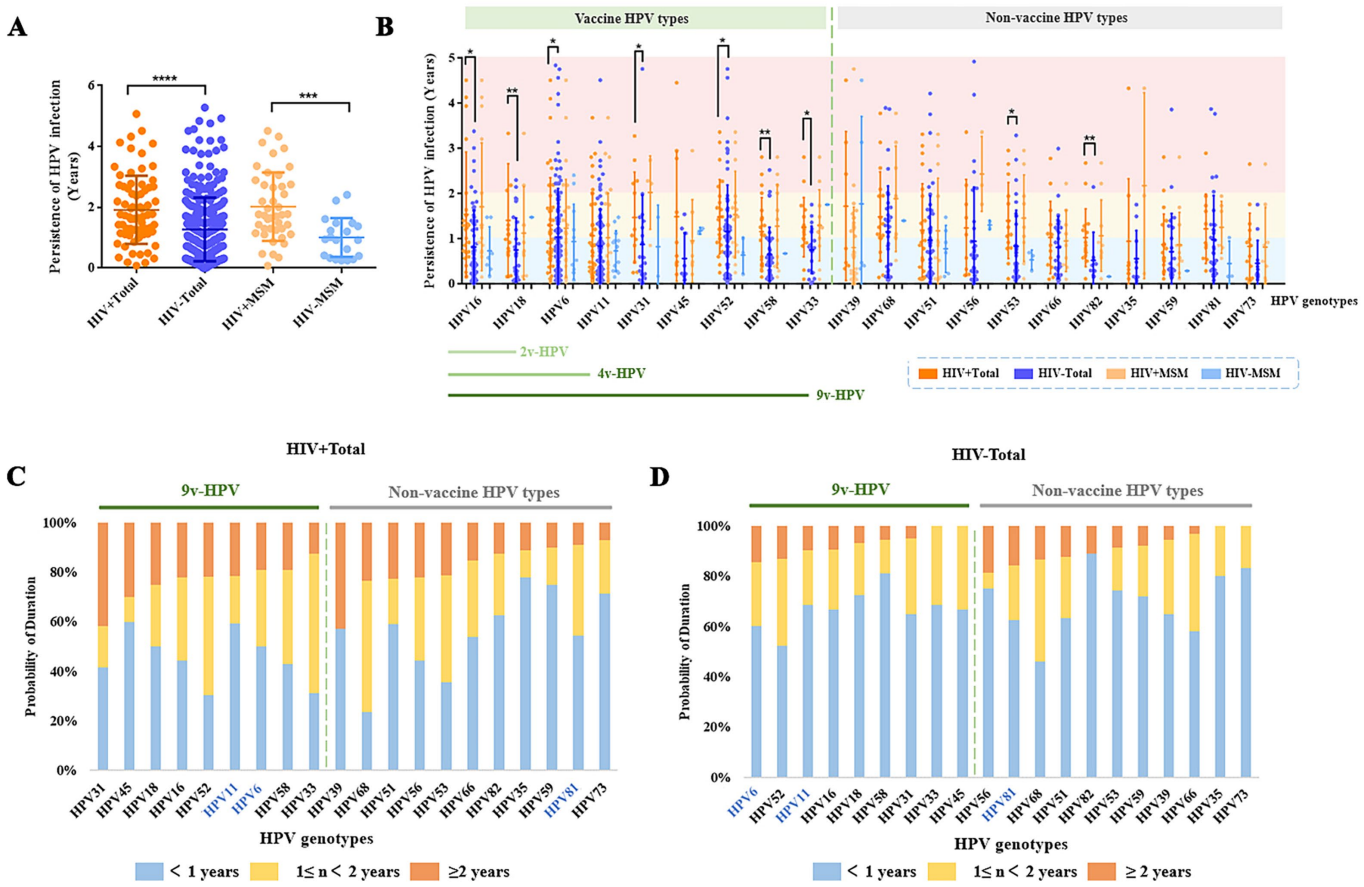
HPV persistence duration among different male patient groups with long-term (≥1 year) follow-up. **(A)** Persistence duration of any HPV type infection in HIV^+^, HIV^−^ populations. **(B)** Duration of vaccine-targeted versus non-vaccine-targeted HPV subtypes in male patients. **(C)** Probability of persistent infection for vaccine-targeted versus non-vaccine-targeted HPV subtypes in HIV^+^ patients. **(D)** Probability of persistent infection for vaccine-targeted versus non-vaccine-targeted HPV subtypes in HIV^−^ patients.

HPV persistence was defined as the time interval between the first positive detection and the last positive detection of the infection in a patient. To minimize the potential influence of therapy duration, we selected 384 male patients with follow-up durations ≥1 year for further analysis. Despite therapeutic intervention, HIV^+^ total males had significantly longer HPV infection than HIV^−^ total males (*p* < 0.05). HPV persistence duration was observed similar between HIV^+^ MSM and the HIV^+^ total males (Average: 2.02 years vs. 1.92 years) (Figure 4A). For certain non-vaccine-related HPV types (specifically HPV53 and HPV82), infection persistence was significantly prolonged in HIV^+^ total males compared to HIV^−^ individuals (*p* < 0.05) (Figure 4B). HIV^+^ total males were more likely to maintain persistent infections ≥2 years with HPV39 (43%), HPV31 (42%), and HPV45 (30%), while HIV^−^ total males showed longer persistence (≥2 years) with HPV56 (19%), HPV81 (16%), HPV68 (14%), and HPV6 (14%) (Figures 4C,D; Supplementary Tables 4, 5). Males over 45 years old showed longer HPV persistence than younger age groups in comparative analysis (Supplementary Table 6).

### Incidence of type-specific HPV infection

**Figure 5:**
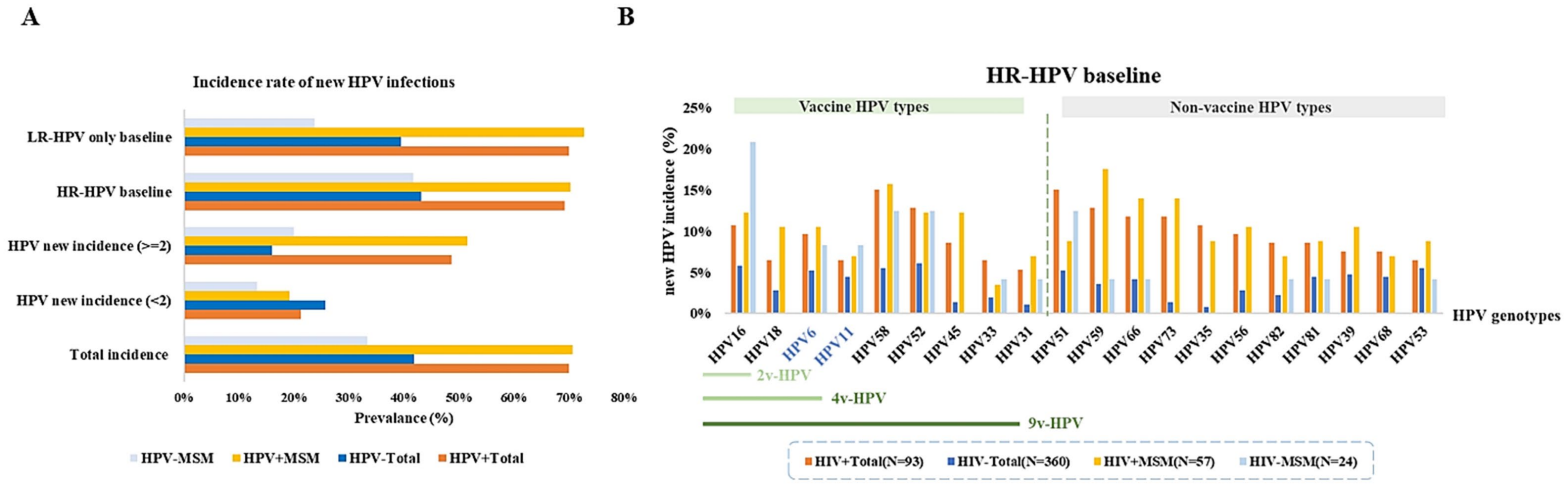
The prevalence of new HPV in patients with HPV infection. **(A)** Proportions of new HPV infections, new multiple-type HPV infections, and probabilities of new infections in patients initially presenting with either HR-HPV or LR-HPV types. **(B)** Probability of acquiring vaccine-targeted HPV types during follow-up among patients with initial HR-HPV infection.

Across all HPV testing results, the detection of a genotype distinct from those found in the baseline test was defined as a new HPV incidence (0–1). HIV^+^ individuals with HPV infection were more susceptible to acquiring new HPV infections. 70% of HIV^+^ total males and 71% of HIV^+^ MSM developed new HPV infections. Among patents with new HPV infection, 70% of HIV^+^ total males and 73% of HIV^+^ MSM acquire two or more new HPV genotypes. In contrast, only 42% of HIV^−^ total male patients and 33% of HIV^−^ MSM develop new HPV infections. Among patients with baseline HR-HPV infection, HIV^+^ individuals exhibit a higher incidence of HPV infections. Similarly, for those with only LR-HPV at baseline, both HIV^+^ total males and HIV^+^ MSM demonstrated significantly higher susceptibility to incident HPV infections (*p* < 0.05) (Figure 5A; Supplementary Table 7). Among patients with baseline HR-HPV infection, HIV^+^ total males were more likely to acquire new HPV infections of types 51, 58, and 52, whereas among HIV^+^ MSM, the most frequently acquired new HPV types were 59, 58, 66, and 73. Notably, the majority of these newly acquired HPV types were not covered by current vaccine^−^specific protection (Figure 5B; Supplementary Table 8).

### Risk modeling of HIV effects on clearance and acquisition of type-specific HPV genotypes

We employed a multi-state Markov model to quantify type-specific transition intensities (TIs) between HPV-uninfected and infected states, and assessed the impact of HIV infection on transitions to incidence and clearance. TIs are reported per person-year at baseline, and the effect of HIV is presented as hazard ratios (HRs) with 95% confidence intervals (95%CIs).

Among vaccine-related HPV types, HIV infection did not significantly alter incidence for HPV16 HPV18, HPV6 and HPV11, but markedly reduced clearance for HPV16 (HR = 0.463; 95%CI: 0.288–0.742) and HPV18 (HR = 0.483; 95%CI: 0.275–0.848), HPV6 (HR = 0.351; 95%CI: 0.239–0.516) and HPV11 (HR = 0.387; 95%CI: 0.261–0.573). For 9v-HPV-relevant types, HIV infection was associated with significantly increased incidence for HPV52 (HR = 1.835; 95%CI: 1.104–3.049), HPV58 (HR = 2.360; 95%CI: 1.357–4.105), and HPV45 (HR = 2.866; 95%CI: 1.140–7.201). Clearance of HPV58 (HR = 0.579; 95%CI: 0.345–0.972), HPV33 (HR = 0.346; 95%CI: 0.180–0.668), and HPV31 (HR = 0.408; 95%CI: 0.181–0.923) was significantly reduced in HIV^+^ individuals.

**Figure 6:**
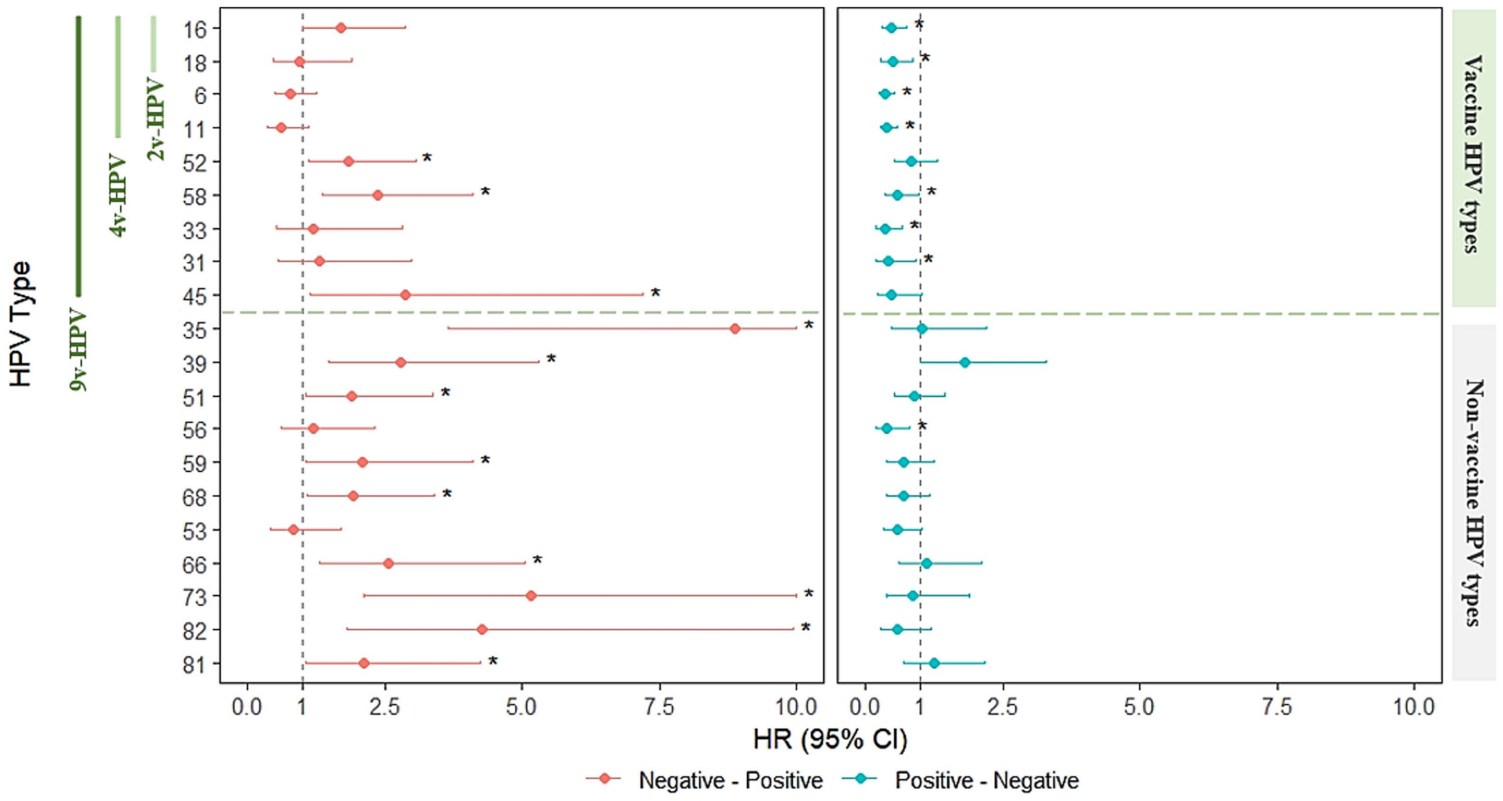
Markov modeling of HIV effects on HPV infection state transitions in males. (Left) HIV-associated HRs for transitioning from uninfected to infected (incidence). (Right) HIV-associated HRs for transitioning from infected to uninfected (clearance). The vertical dashed line across HR = 1 indicates no statistically significance in HIV infection. Asterisks (*) indicate statistical significance (*p* < 0.05).

For non–vaccine-relevant HPV types, TIs from uninfected to infected were generally low, but HIV infection substantially increased incidence for nearly all HPV types, including HPV35 (HR = 8.880; 95%CI: 3.657–21.560), HPV39 (HR = 2.789; 95%CI: 1.468–5.298), HPV51 (HR = 1.882; 95%CI: 1.052–3.365), HPV59 (HR = 2.081; 95%CI: 1.055–4.104), HPV68 (HR = 1.915; 95%CI: 1.076–3.408), HPV66 (HR = 2.560; 95%CI: 1.297–5.052), HPV73 (HR = 5.163; 95%CI: 2.116–12.595), HPV82 (HR = 4.251; 95%CI: 1.819–9.936), and HPV81 (HR = 2.127; 95%CI: 1.065–4.248). Clearance was significantly reduced for HPV56 (HR = 0.394; 95%CI: 0.196–0.796), while other types showed variable, mostly non-significant, HIV-related effects on clearance (Supplementary Table 9; Figure 6).

## Discussion

Globally, the estimated overall HPV prevalence among men is 31% (95% CI, 27–35) (6), while MSM, particularly those co^−^infected with human immunodeficiency virus (HIV), exhibit significantly higher HPV infection rates (16–19). In China, the most prevalent HPV genotypes among MSM include HPV6, 11, 16, 18, 52, and 58 (20). The HPV genotypes detected most frequently were HPV6, 16, 11,18, 58, and 52 in Shenzhen (18). In our study, the most prevalent HR-HPV types among HIV^+^MSM included HPV11, 6, 52, 16, 51. The most common HPV types in MSM populations are largely similar, though regional differences exist. HPV16 was the most prevalent type in anal samples (19.0%), followed by HPV6 (18.5%), HPV51 (16.4%), and HPV52 (12.8%) in MSM in France (21). Therefore, it is necessary to conduct epidemiological surveys on HPV among MSM populations in different regions to evaluate the preventive effectiveness of the HPV vaccine.

The duration of HPV persistent infection also indicates its clearance status from another angle in our study. Natural history studies have consistently demonstrated approximately half of HPV infections persist past 6 to 12 months (22) and lower clearance rates for HR-HPV infections compared to LR-HPV (23). HIV infection increases the risk of HPV persistent infection for the immune response suppression in patients (24, 25). HIV^+^MSM had a higher persistence of HPV16 than HIV^−^ MSM (16.7% vs. 1.3%, *p* < 0.001) (26). A 12-month follow-up study showed reported that the five HPV genotypes responsible for the most persistent infections among MSM were HPV6, 39, 58, 33, and 51 in China (27). We observed significantly extended HPV infection persistence in male individuals above 45 years of age compared to younger groups. The broader sexual history and greater cumulative partner count typically seen in older males were related to lower HPV clearance rates (27). In our study, HIV^+^ total males remained significantly more prone to persistent infections (≥2 years)with HPV39, 31, 45, whereas long^−^term infections in HIV^−^ total males were predominantly associated with HPV56, 81, 68 and HPV6. Notably, multiple non-vaccine-targeted HPV types persisted for over 1–2 years in both HIV^+^ and HIV^−^ groups, highlighting the substantial limitations of current vaccines in covering HPV variants. Even after HPV vaccination, patients may still experience prolonged HPV infections.

Population^−^based studies have provided valuable insights into HPV infection dynamics. Previous research has reported that the Incidence rates (IRs) of anal infections in MSM had a median of 5.2 per 100 person-years (range: 2.2–7.9) across types, with HPV16 having the highest IR (15). HPV incidence was approximately double among HIV^+^ population compared to those without (28). In this study, the Markov model indicated no significant association between HIV and the occurrence of multiple HPV types, suggesting that HIV is not a major contributing factor to the incidence of these HPV infections. Lower education levels, multiple sexual partners, and younger age have also been reported to be associated with a higher incidence of HPV infection in MSM (15, 27, 29). Clinical trials have demonstrated that the HPV vaccine provides durable protection against anogenital diseases in both heterosexual men and MSM populations. However, breakthrough HPV infections may still occur in vaccinated individuals (30–32). As reported by Lepiller et al., HR-HPV, 2v-HPV, 4v-HPV, and 9v-HPV were detected in 65.6, 24.9, 43.7, and 58.0% of anal samples in MSM, respectively (21). In our study, approximately 2/3 of HIV^+^ and 2/5 of HIV^−^ males were infected with HR-HPV covered by the 9v-HPV. These findings suggested that the majority of HPV infections in the male population were covered by the 9-valent vaccine, demonstrating its potential efficacy in preventing HPV-related disease among men. Therefore, HPV vaccination is recommended for the males, particularly for HIV^+^ individuals. Meanwhile, HPV types not currently covered by vaccines should also be considered in future research and development.

This study has several methodological limitations that warrant consideration. First, this study enrolled male patients who visited the hospital and were diagnosed with HPV infection. Most of these patients performed HPV testing due to reasons such as “high-risk sexual behaviors,” “presentation of typical clinical symptoms,” or “having sexual partners with STDs,” thus being identified as a high-risk population for HPV. Therefore, the observed incidence of different HPV genotypes were higher than that in the general male population. Second, due to privacy concerns, some patients declined to disclose their history of male-to-male sexual contact, making it difficult to definitively classify their MSM status; consequently, we were unable to conduct HPV epidemiological analyses specifically for non-MSM subgroups. Furthermore, our institution serves as a designated HIV treatment center, resulting in an overrepresentation of HIV^+^ individuals that may compromise the generalizability of both HPV and HIV infection rate estimates. Importantly, the statistical results in this study were influenced by medications for HPV and HIV, and thus do not reflect the natural history of HPV persistence and clearance. The duration and frequency of patient follow^−^ups were primarily determined by voluntary healthcare^−^seeking behaviors and HPV treatment outcomes, which may introduce variability in the assessment of HPV persistence duration. These factors should be carefully considered when interpreting our findings.

## Data Availability

The raw data supporting the conclusions of this article will be made available by the authors, without undue reservation.
