# Supplementary material for: Incidence and persistent infection rates of type-specific HPV among HIV-infected males in China: a 5-year retrospective study
Source: Front Public Health. 2025 Nov 5;13:1690348. doi: 10.3389/fpubh.2025.1690348 (PMC12627005; doi:10.3389/fpubh.2025.1690348)
Supplement: Supplementary file 1 [file Table_1.docx]

**Supplemental Table 1.** Distribution of HPV^+^patients by age group at baseline.

| **Age (years)** | **Total (N=756)** | | ***P*-value (*Fisher's test*)** | **MSM（N=113）** | | ***P*-value (*Fisher's test*)** |
| --- | --- | --- | --- | --- | --- | --- |
|  | **HIV^+^(N=123)** | **HIV^-^(N=633)** |  | **HIV^+^(N=68)** | **HIV^-^(N=45)** |  |
|  |  |  | 0.067 |  |  | 0.138 |
| ＜20 | 4 (0.03) | 14 (0.02) |  | 4 (0.06) | 2 (0.04) |  |
| 20~26 | 28 (0.23) | 143 (0.23) |  | 14 (0.21) | 18 (0.40) |  |
| 27~35 | 64 (0.52) | 266 (0.42) |  | 34 (0.50) | 21 (0.47) |  |
| 36~45 | 18 (0.15) | 162 (0.26) |  | 10 (0.15) | 3 (0.07) |  |
| ＞45 | 9 (0.07) | 48 (0.08) |  | 6 (0.09) | 1 (0.02) |  |
|  |  |  |  |  |  |  |

**Supplemental Table 2.** Prevalence of HPV types in HIV^+^ and HIV^-^ male and MSM.


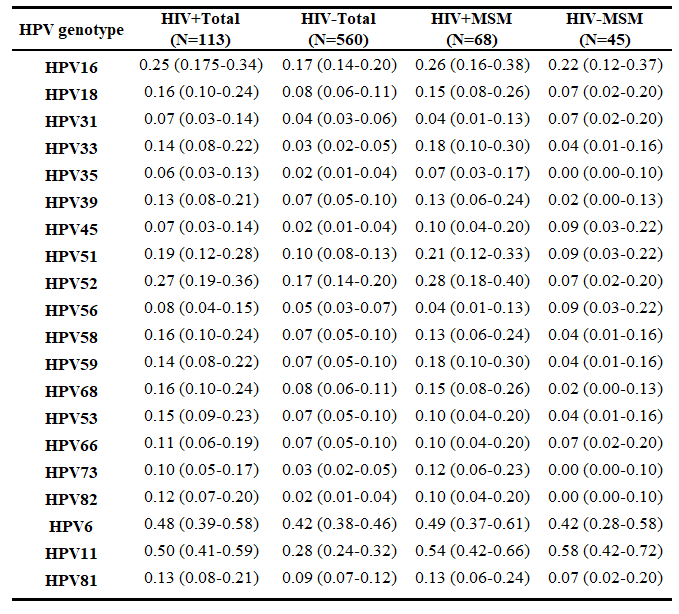


**Supplemental Table 3.** Prevalence of vaccine-targeted HPV types in male.


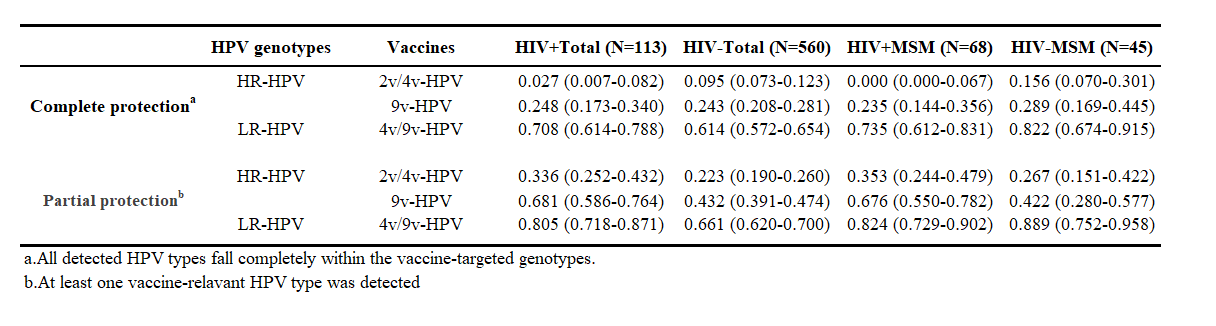


**Supplemental Table 4.** Duration of type-specific HPV persistence in HIV-positive patients receiving long-term follow-up (≥1 year).


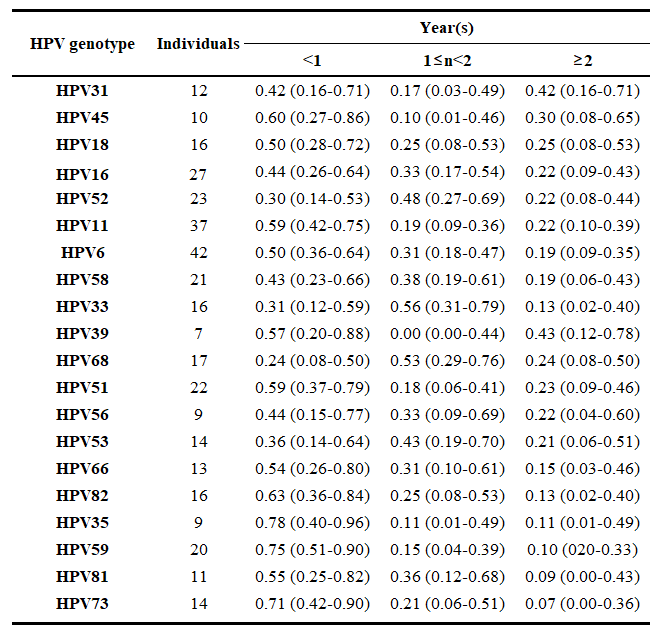


**Supplemental Table 5.** Duration of type-specific HPV persistence in HIV-negative patients receiving long-term follow-up (≥1 year).


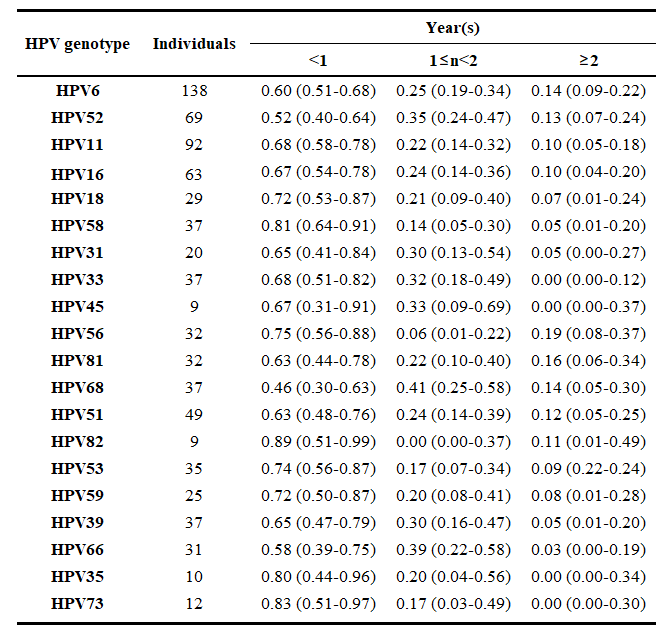


**Supplemental Table 6.** Average duration of males by age with long-term follow-up (≥1 year).


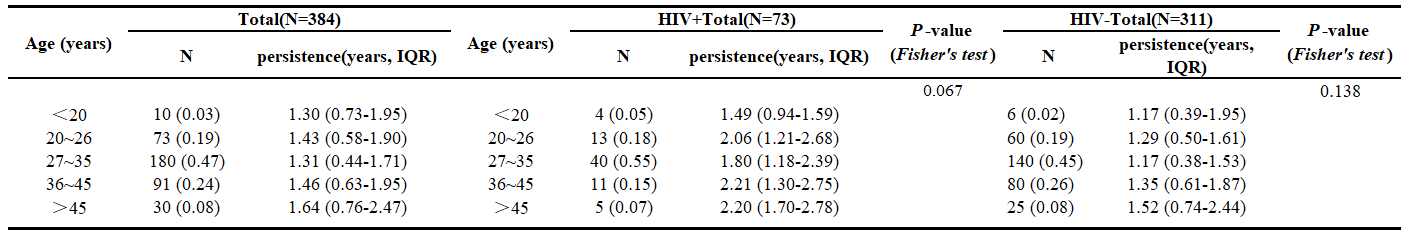


**Supplemental Table 7.** Incidence of new HPV infections among HPV^+^ individuals


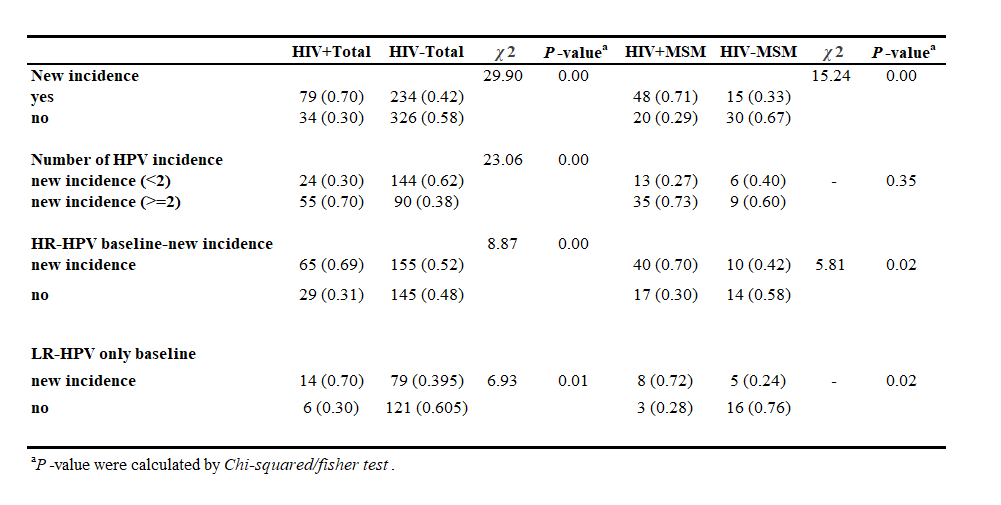


**Supplemental Table 8.** Incidence of HPV infection during treatment among patients initially diagnosed with high-risk HPV(HR-HPV).


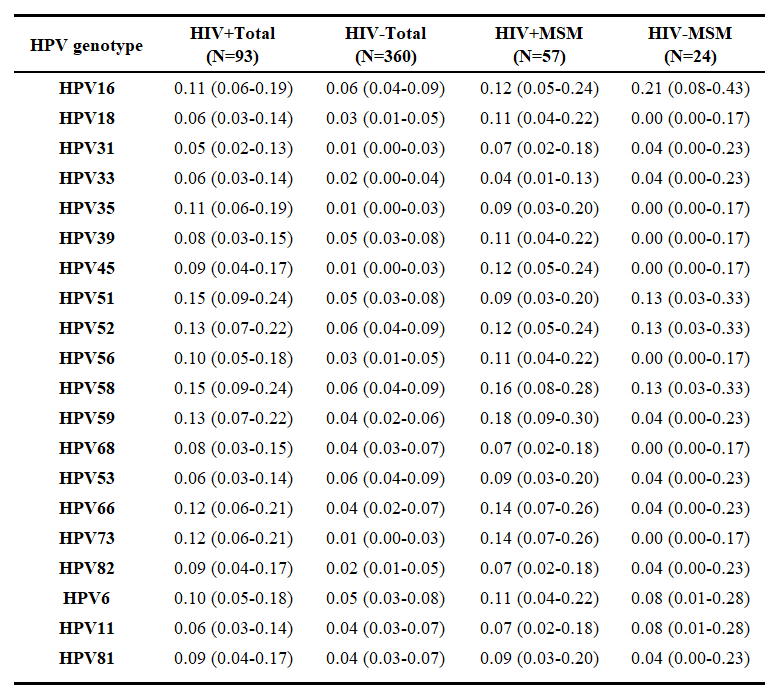


**Supplemental Table 9.** **Markov modeling of HIV effects on HPV infection state transitions in males.**
